# Sphingolipid metabolism in T cell responses after allogeneic hematopoietic cell transplantation

**DOI:** 10.3389/fimmu.2022.904823

**Published:** 2022-08-16

**Authors:** Linlu Tian, Besim Ogretmen, Brian Y. Chung, Xue-Zhong Yu

**Affiliations:** ^1^Department of Microbiology & Immunology, Medical College of Wisconsin, Milwaukee, WI, United States; ^2^Department of Biochemistry & Molecular Biology and Hollings Cancer Center, Medical University of South Carolina, Charleston, SC, United States; ^3^The Cancer Center, Medical College of Wisconsin, Milwaukee, WI, United States

**Keywords:** sphingolipid metabolism, graft versus host disease, graft versus leukemia response, allogeneic hematopoietic cell transplantation, T cell

## Abstract

Allogeneic hematopoietic cell transplantation (allo-HCT) is an effective immunotherapy against hematopoietic malignancies. The infused donor lymphocytes attack malignant cells and normal tissues, termed a graft-verse-leukemia (GVL) effect and graft-verse-host (GVH) response or disease (GVHD), respectively. Although engineering techniques toward donor graft selection have made HCT more specific and effective, primary tumor relapse and GVHD are still major concerns post allo-HCT. High-dose systemic steroids remain to be the first line of GVHD treatment, which may lead to steroid-refractory GVHD with a dismal outcome. Therefore, identifying novel therapeutic strategies that prevent GVHD while preserving GVL activity is highly warranted. Sphingolipid metabolism and metabolites play pivotal roles in regulating T-cell homeostasis and biological functions. In this review, we summarized the recent research progress in this evolving field of sphingolipids with a focus on alloreactive T-cell responses in the context of allo-HCT. We discussed how sphingolipid metabolism regulates T-cell mediated GVH and GVL responses in allo-HCT and presented the rationale and means to target sphingolipid metabolism for the control of GVHD and leukemia relapse.

## Introduction

Pioneered by E. Donnell Thomas and colleagues in the early 1960s (1, 2), hematopoietic cell transplantation (HCT) is performed on approximately 50,000 patients annually worldwide as a treatment for hematological malignancies (*e.g.*, myeloma, lymphoma, leukemia, and myeloproliferative neoplasms) and nonmalignant conditions (*e.g.*, sickle cell disease, inherited bone marrow failure syndromes, transfusion-dependent thalassemia, inherited immune deficiency syndromes, and certain metabolic disorders) (2, 3). In recent years, the HCT treatment is being increasingly personalized and the donor selection process follows strict rules that aim to weight the need for limiting disease relapse versus toxicity, which requires a careful choice between bone marrow and growth factor-mobilized peripheral blood from HLA match, unrelated, haploidentical or infrequently umbilical cord blood donors (4). Conditional regimens (irradiation or chemotherapy) and hematopoietic cell products obtained from donors exhibit graft-versus-tumor (GVT) response. While the donor alloimmunity towards recipient malignant cells limits or prevents relapse, it also attacks normal tissues and gives rise to graft-versus-host disease (GVHD) (5).

More than one million hematopoietic cell transplants have been performed to date, 40% of which were allogeneic (3, 6). The most common life-threatening complication associated with allogeneic-HCT (allo-HCT) is GVHD. GVHD develops in two forms: acute and chronic with distinct etiology, pathophysiology, and response to therapeutic regimens. Although patients who undergo allo-HCT can benefit from graft-versus-leukemia (GVL) effects, GVHD, which is closely linked to GVL response, is a significant cause of morbidity and mortality after transplantation. Clinically severe GVHD often leads to organ injury, poor quality of life, secondary malignancies, or opportunistic infections. The high incidence of morbidity and mortality suggests that GVHD remains an “unsolvable” complication in many post-transplant patients (7, 8).

As a leading cause of mortality after allo-HCT, latent bacterial, viral, and fungal infections are frequent (9). To some extent, prophylactic and preemptive pharmacotherapies are limited by the toxicity and the lack of efficacy in breakthrough infections. The reconstitution of infused donor T cells plays a vital role in effective infection control following allo-HCT (10). Although donor T cells contribute to engraftment and to protection of patients from opportunistic infections and residual diseases, they can also induce severe GVHD. The HCT donors are selected from bone marrow or growth factor-mobilized peripheral blood from HLA-matched, haploidentical or infrequently umbilical cord blood donors. T cells are sometimes depleted from donor grafts to attenuate the risk of T cell-driven GVHD (11). As a result, it takes many months to reach functional recovery of donor-derived T cells (12), increasing the risk of fatal infection (13). However, without T-cell completed depletion in allografts, donor-derived T cells can engage antigen-processing cells (APCs) from either the host or the donor, promoting their activation and proliferation. Donor-activated CD4^+^ T cells differentiate into T helper (T) cells Th1, Th2, Th9, Th17, Th22, or regulatory T cells (Tregs), while CD8^+^ T cells differentiate into cytotoxic T cells. These differentiated T cells migrate to target organs and mediate both GVH and GVL responses (14, 15). Targeted regulation of alloreactive donor T-cell activation, cytokine production, and cell migration are a few promising strategies that can impede GVHD pathogenesis. The optimal therapeutic approach would be to control GVHD development while maintaining, or even augmenting, GVL effects upon allo-HCT.

Sphingosine and its relatives compose the backbone of eukaryotic lipids. Sphingolipids, including ceramide, sphingomyelin, and different glycosphingolipids, are profoundly important to cell function and pathology (16). The sphingolipid metabolites ceramide, ceramide-1-phosphate (C1P), and sphingosine-1-phosphate (S1P) are crucial signaling molecules that control immune cell trafficking and fate, which have implications for immune-related diseases and antitumor immunity, including GVHD (17). In this review, we summarize recent discoveries in the evolving field of sphingolipid metabolism as it relates to T-cell responses in the context of allo-HCT. We also discuss how these findings can help us reevaluate our current understanding of immune response in allo-HCT, which can help shaping novel immunotherapeutic strategies that promote long-term tolerance in transplant recipients while controlling GVHD and leukemia relapse.

## Overview of the sphingolipid signaling pathway

**Figure 1:**
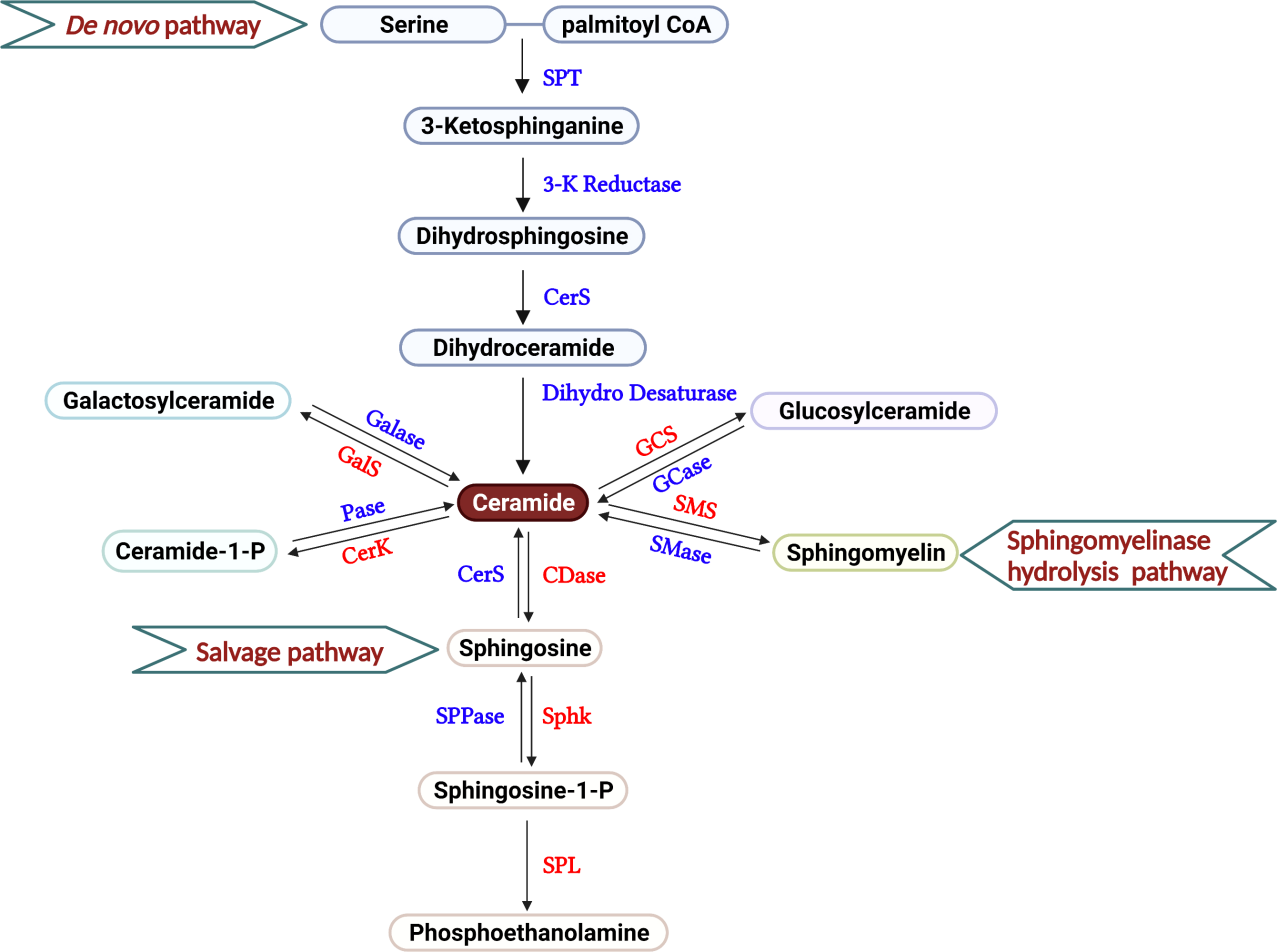
Overview of sphingolipid metabolism. The three different pathways that produce the sphingolipids in T cells are described, including *de novo* generation, salvage, and sphingomyelinase hydrolysis pathways. In the current review, the roles of galactosyl ceramide, ceramide and S1P are primarily discussed in the regulation of T cell-mediated GVH and GVL responses after allo-HCT. Serine Palmitoyl Transferase (SPT), 3-Ketosphinganine Reductase (3-K Reductase), Ceramide Synthase (CerS), Dihydroceramide Desaturase (Dihydro Desaturase), Ceramidase (CDase), Sphingosine Phosphate Phosphatase (SPPase), Sphingosine kinase (Sphk), S1P Lyase (SPL), Galactosyl ceramidase (Galase), Galactosylceramide synthase (GalS), Phosphatase (Pase), Ceramide Kinase (CerK), Glucosyl ceramidase (GCase), Glucosylceramide synthase (GCS), Sphingomyelinase (SMase), Sphingomyelin synthase (SMS).

Ceramides, composed of fatty acids and sphingosines, are structural members of the cell membrane. Three major pathways are thought to supply the cell with ceramides, including *de novo* generation, sphingomyelin hydrolysis, and the salvage pathway (**Figure 1**). In the endoplasmic reticulum (ER), serine palmitoyltransferase (SPT) facilitates the condensation of palmitoyl coenzyme A (CoA) and serine to form 3-ketosphinganine, the rate-limiting step of this *de novo* pathway (18). By 3-ketosphinganine reductase, 3-ketosphinganine rapidly converts into dihydrosphingosine, which is then N-acylated by ceramide synthases (CerS1–CerS6) to generate dihydroceramide, using saturated or monounsaturated fatty acids containing 14 to 26 carbons. Dihydroceramides are subsequently dehydrogenated into ceramides *via* dihydroceramide desaturases (19). Ceramides are also supplied through the sphingomyelin hydrolysis pathway, mainly through the activation of sphingomyelinases (SMases) in the Golgi (20). Ceramide kinase phosphorylates ceramides to form ceramide-1-phosphate (C1P), which are then deacylated by ceramidase to generate sphingosine (21). By catalyzing with glucosylceramide synthase and galactosylceramide synthase, ceramide can be assembled into glucosylceramide and galactosylceramide. Sphingolipid metabolism exhibits rapid turnover, and the balance between sphingolipids and their metabolites are controlled by synthesis and degradation reactions across multiple compartments (22). In the salvage pathway, ceramide synthase acylates a significant amount of sphingosine molecules to generate ceramide, which helps maintain sphingolipid homeostasis within the cell (22, 23). Moreover, ceramide can also be recycled from C1P through phosphatase, glucosylceramide by glucosylceramidase, or galactosylceramide *via* galactosylceramidase.

Sphingosines are phosphorylated by two isoforms of sphingosine kinase, SphK1 and SphK2, to generate sphingosine-1-phosphate (S1P) (24). S1P produced by Sphk2 localizes in the cell nucleus and, to a lesser extent, the mitochondria (25, 26). In contrast, S1P generated by Sphk1 is primarily confined to the cytoplasm or secreted into the extracellular matrix (27). S1P can either be dephosphorylated by S1P-specific phosphatases, SPP1 and SPP2, or irreversibly degraded by S1P lyase (SPL) to phosphoethanolamine (28) (**Figure 1**). Various stimuli, such as pathogens, oxidative stress, etc. influence cell sphingolipid metabolism *via de novo* generation, sphingomyelin hydrolysis, and the salvage pathways spatially and temporally, leading to the production of specific bioactive metabolites (22). Better understanding the action of sphingolipid metabolism and its metabolites on T-cell response may provide rationale and novel strategies that separate GVH and GVL responses.

## Ceramide metabolism

### *De novo* generation

*De novo* ceramide biosynthesis, which occurs in the ER and mitochondrion, is critical for producing ceramides within the cell. CerS1-CerS6 introduce various acyl-CoA side chains to the sphingosine backbone in a length-specific manner (29). CerS1 isoform primarily synthesizes C18-ceramide (30), CerS4 generates C18-/C20-ceramide (31), CerS5 and CerS6 share an overlapping specificity for C14-/C16-ceramide (31, 32), CerS2 utilizes very-long acyl chain CoAs to produce C22-/C24-ceramide (33), and CerS3 yields C26-ceramide and above (29). Interestingly, the biological functions of these ceramides with different fatty acyl chain lengths are also very distinct, dependent on the cellular context, subcellular localization, and their downstream target proteins.

In the murine study, CD4^+^ T cells increase the transcription of CerS2, CerS4, CerS5 and CerS6 when stimulated with anti-CD3/CD28 antibodies while CD4^+^ T cells from CerS2 null mice show lower levels of these CerSs than WT cells (34). CerS2 deficiency enhances TCR signal strength as compared to WT control in activated CD4^+^ T cells, which is reflected by increased phosphorylation of extracellular-regulated kinase (ERK) and AKT. The increased TCR signal induced by CerS2 deficiency further promotes the differentiation of CD4^+^ T cells towards Th17, but away from Th2 (34). Different from the proinflammatory responses of CerS2 knockout in CD4^+^ T cells, transfer of CerS6-deficient CD4^+^ T cells induced less colitis compared to WT cells (35). However, C14/C16-ceramides generated by CerS6 in the mitochondria lead to mitophagy dysfunction in activated T cells, inducing aging-dependent metabolic disorders that decreased T-cell central memory phenotype and reduced antitumor immunity in aged mice (36). Germline loss of CerS5 or CerS6 exacerbates inflammation in a dextran sulfate sodium (DSS)-induced colitis mouse model (37, 38). These findings demonstrate that CerS isoenzymes play different roles in modulating T-cell biological functions.

Post transplantation, C16-ceramides generated by CerS6 promote donor T-cell expansion, migration, and differentiation into Th1/Tc1, but away from Treg, further inciting GVHD in both major histocompatibility complex (MHC) mismatched and haploidentical bone marrow transplantation (BMT) mouse models (39). In contrast, CerS4 had a minimal effect on T-cell alloresponse and pathogenicity in on GVHD (39). Mechanistic studies revealed that CerS6/C16-ceramide is vital for optimal T cell receptor (TCR) signal transduction *via* Zap-70, triggering tyrosine phosphorylation and colocalization of CD3 and protein kinase θ (PKCθ) in CD4^+^ T cells. However, CerS6/C16-ceramide is largely dispensable for Zap-70 mediated TCR signal transduction on CD8^+^ T cells. Further, treatment with a CerS4/S6 inhibitor ST1072 at 2mg/kg or less significantly reduced GVHD while presenting little or no toxicity and preserving the GVL activity (40, 41). Both genetic deletion and pharmacological inhibition of CerS6 preferentially impair the alloreactivity of CD4^+^ T cells over CD8^+^ T cells. For translational purpose, the xenograft models with or without Raji lymphoma infusion are employed to validate the efficiency of ST1072, wherein HLA-A2^–^ human PBMCs are transplanted into irradiated NSG-A2^+^ mice. Consistent with the results from murine allogenic and haploidentical BMT models, the recipient NSG-A2^+^ mice treated with ST1072 markedly decrease GVHD development and tumor relapse (41). These findings provide a strong rationale for targeting CerS6 to control GVHD while preserving the GVL activity.

Only scarce reports can be found in studying the role of CerS in human T cells. CD4^+^ Jurkat cells enhance the transcription of all the CerSs except for CerS4 when stimulated with anti-CD3 antibody and IL-2, while silencing CerS5 by shRNA prohibits the upregulation of CerS1, CerS3 and CerS6 and significantly decreases CerS4 transcription upon stimulation (38). CerS5 deficiency in human CD4^+^ Jurkat cells impairs NF-κB dependent T-cell signal and proper T-cell activation (38). After HCT, pharmacological inhibition of CerS6 with ST1072 reduces the numbers of IFN-γ-producing and total human T cells in secondary lymphoid and GVHD target organs in a xeno-GVHD model (41). The concentration of C16-ceramide obviously increases in the serum of patients with GVHD than those without GVHD post HCT (41). Since CerS5 and CerS6 have overlapping specificity in producing Cer14- and Cer16-ceramides, distinct or redundant roles of these two isoforms in T cells remain to be studied. We interpret that inhibition of CerS6 with ST1072 might indirectly affect the level of CerS5, which may in turn impact T-cell response and GVHD pathogenicity. The combined blockades of CerS5 and CerS6 may be more effective in the prevention of GVHD development.

Taken together, pre-clinical studies so far demonstrate that CerS6 is a potential therapeutic target for the prevention or treatment of GVHD after allo-HCT. Given abundant expression of the other CerS isoenzymes in T cells upon activation, their roles in T-cell response deserve further investigation. To translate pre-clinical findings to clinical application, the role of individual CerSs in human T cells and GVHD development should be further investigated; the development of low toxic and more specific inhibitors of CerS will be required.

### Sphingomyelinase hydrolysis generation

In addition to *de novo* biosynthesis, the sphingomyelinase hydrolysis generation pathway produces ceramide from sphingomyelin through acid or neutral sphingomyelinase (ASMase or NSMase, respectively). This pathway can produce ceramides rapidly when required. In human T cells, ceramides generated from sphingomyelins can upregulate TCR and impact TCR recycling dynamics in a concentration- and time-dependent manner, thus influencing T cell responsiveness (42). Mouse T cells treated with either exogenous sphingomyelinase or a ceramide analog (C6-ceramide) imitate CD28 signaling, promoting both T cell proliferation and IL-2 gene transcription (43). In both human and murine T cells, the enhancement of sphingomyelinase hydrolysis generation pathway arguments T-cell responses.

Genetic deletion of ASMase in mice causes an increase in Tregs among CD4^+^ T cells and limited IL-17 production (44). Reducing ceramide generation by depleting ASMase does not impact IL-2 or IL-2 receptor expression but decreases IL-2 secretion. The reduction of IL-2 generation further impairs T-cell proliferation induced by anti-CD3/anti-CD28 or concanavalin A (con A) (45). Similar observations are made in human CD4^+^ T cells: pharmacological inhibition of ASMase significantly attenuates proliferation of human CD4^+^ T cells induced by anti-CD3/anti-CD28 stimuli (46). Although cytolytic granules of ASMase knockout and wild-type CD8^+^ T cells are equally loaded with granzymes and perforin, deficiency for ASMase in CD8^+^ T cells markedly impairs the contraction of secretory granules (47). Ligation of Fas on activated T cells stimulates ASMase activity, leading to ceramide production and, consequently, induces cell death (48, 49).

In an MHC mismatched murine model where allogeneic bone marrow and splenic T cells are transplanted into ASMase^+/+^ and ASMase^-/-^ recipients, host ASMase is required for full-blown acute GVHD. Specifically, loss of ASMase reduces acute inflammation, cytokine storm, CD8^+^ T-cell alloreactivity, diminishes GVHD target organs injury (liver, intestinal, and skin), and improves recipient morbidity and mortality after allo-HCT (50).

Ceramides generated by the *de novo* and sphingomyelinase hydrolysis pathways contribute to cytokines production, activation, or proliferation of human or murine T cells. Deficiency of either CerS6/C16 ceramide in donor T cells (39) or ASMase/ceramide (50) in recipients prevents GVHD development. Genetic deletion and pharmacological inhibition of CerS6 have negligible effect on donor CD8^+^ T cells, thus maintaining GVL response post HCT. However, the deficiency for ASMase in CD8^+^ T cells attenuates the contraction of secretory granules and alloresponse after allo-HCT, which indicate a possible impairment of CTL activity. Therefore, the impact of ASMase/ceramide on GVL response remains to be determined (**Table 1**).

**Table 1:** The impacts of targeting sphingolipids in GVH and GVL responses after allo-HCT.

Intervention	GVH	GVL	References
No intervention	+++	+++	N/A
Inhibition of CerS6	+	++	(39, 41)
Inhibition of ASMase	+	?	(50)
Administration of α-galactosyl ceramide	+	++	(51–55)
Inhibition of S1P/S1PRs	+	+	(41, 56–63)

+ weak, ++ moderate, +++ strong, ? unknown.

## Glycosphingolipid metabolism

The glycosphingolipid, galactosylceramide has been regarded as a ligand of invariant natural killer T (iNKT) cells and is responsible for host defense. Unlike conventional T cells, which can recognize peptides presented by MHC, iNKT cells are defined as unconventional T cells and restricted by monomorphic MHCs. This subset of T cells expresses semi-invariant TCRs, limiting the antigen recognition range analogously to innate immune receptors (64). As a unique subset of αβT cells, iNKT cells play a pivotal role in modulating GVH and GVL response (51, 65). In human studies, the recovery of donor derived iNKT cells early post-transplantation presents the association with the reduced non-relapse mortality (66). The ratio of iNKT/T cells and CD4^-^ iNKT-cell dose in donor bone marrow and peripheral blood stem cell grafts appear as independent predictors of the occurrence and risk of aGVHD post HCT (66–68). iNKT cells can significantly attenuate the expansion capacity of T cells and a higher dose of iNKT cells in allografts correlates with an improved GVHD- and progression-free survival after allo-HCT (69).

α-galactosyl ceramide, a prototypical iNKT cell ligand, was first extracted from the marine sponge *Agelas Mauritians* (70). In the mouse model of MHC-mismatched BMT, α-galactosyl ceramide promotes iNKT cell activation in a CD1d-restricted manner, shifts Th1 cytokine production into the Th2 cell type, and attenuated GVHD (51). Moreover, the recipient mice administered with the α-galactosyl ceramide derivative RGI-2001 at transplantation improves GVHD by expanding donor-derived CD4^+^ Foxp3^+^ Tregs in the bone marrow and secondary lymphoid organs in a dose-dependent manner (52). Similar outcomes are observed in a recent clinical trial designed to evaluate the safety, tolerability, and pharmacological profile of RGI-2001 when added to standard of care GVHD prophylaxis in patients underwent allo-HCT (53). A subset of patients treated with RGI-2000 exhibit an increase in the number of Ki-67^+^ Helios^+^ Foxp3^+^ cells, suggesting that α-galactosyl ceramide can expand natural Tregs in allo-HCT patients (53). In a haploidentical HCT mouse model, administration with a reduced dose of cyclophosphamide (PTC) followed by α-galactosyl ceramide induces an NKT2 rather than NKT1 phenotype and early recovery of CD4^+^ Foxp3^+^ Tregs, which prevents GVHD while maintaining GVL effects (54). However, administration of KRN7000, another α-galactosyl ceramide derivative promotes dendritic cell (DC)–dependent NK and conventional T-cell activation and unexpected proinflammation cytokines IFN-γ/TNF-α production that leads to hyperacute GVHD in multiple murine HCT models (55). The factors accounted for such a difference are unknown. It is possible that α-galactosyl ceramide and α-galactosyl ceramide derivative KRN7000 have different structure and activity. Alternatively, administration in combination with PTC might impact the effect of α-galactosyl ceramide.

These studies demonstrate that α-galactosyl ceramide, as a ligand of iNKT cells, provides a reciprocal balance between GVH and GVL responses (**Table 1**). Furthermore, α-galactosyl ceramide and α-galactosyl ceramide derivatives contribute to Treg recovery after allo-HCT, which facilitates to GVHD prevention. The murine studies and clinical trial provide the rationale for clinical translation. However, a better understanding of how various α-galactosyl ceramide derivatives impinge upon associated mechanisms will be necessary to select the most appropriate molecules as therapeutic targets.

## S1P metabolism

### S1P gradient and S1PRs

Hematopoietic and vascular endothelial cells are the primary sources of blood S1P, while lymph S1P is mainly produced by lymphatic endothelial cells (71). Due to the high activity of the S1P-degrading enzyme in tissues, S1P concentrations remain high in blood and lymph and low in tissues. S1P can be exported out of endothelial cells by sphingolipid transporter Spns2 (72), which creates an S1P gradient is important for immune cell trafficking and homeostasis. Vertebrates possess five S1P receptors (S1PR1–5) that respond to extracellular S1P in the hematopoietic system and multiple organs (73). In the mouse thymus, mature single-positive thymocytes upregulate S1PR1 expression and internalization (74). S1PR1 deficiency reduces the emigration of mature thymocytes out of the thymus and results in the accumulation of T and B cells in the second lymphoid organs (74). Cell-autonomous S1PR1 maintains mitochondrial contents by increasing PINK1 levels and inhibiting apoptosis in naïve T cells (75). In human and murine immune systems, S1PR2 can regulate lymphatic endothelial cell (LEC) layer structure and permeability and increase junction molecules expression through the ERK pathway. S1PR1 and S1PR4 separately mediate T-cell motility and vascular cell adhesion molecule-1 (VCAM-1) binding. The incorporation of S1PR1 and S1PR4 on CD4^+^ T cells with S1PR2 on LECs facilitates CD4^+^ T-cell recruitment to LEC migration sites and advanced transcellular migration (76). S1P causes the opposite effects on human naïve and memory T-cell migratory responses. In an S1PR2-dependent manner, S1P can inhibit spontaneous or chemokine-induced migration of memory T cells, which is more pronounced in CD4^+^ than CD8^+^ T cells (77). The interaction of S1P gradient and S1PRs plays a vital role in mediating T-cell trafficking and homeostasis.

Furthermore, S1P signaling can switch T cell differentiation under specific contexts. IL-6/JAK (Janus Kinase)/STAT3 signal transduction, a crucial signaling pathway that is aberrantly hyperactivated in cancer cells or under chronic inflammation conditions, can be directly activated by S1PR1 and feedback onto S1PR1 to prevent its phosphorylation in CD4^+^ T cells, leading to enhanced Th17 polarization (78, 79). The Akt-mTOR kinase pathway, which acts downstream of IL-2 and restrains iTreg generation, can be selectively activated by S1PR1 to further attenuate differentiation of thymic Treg precursors and function of mature Tregs and impact Treg-mediated immunosuppressive ability (80). Smad3 is a crucial signal transducer that modulates TGF-β effects on iTreg generation. S1PR1 signaling controls T-cell lineage specification through mTOR and antagonizes TGF-β mainly by impeding Smad3 activity. These events inhibit the generation of extrathymic and natural Tregs while driving Th1 development in a reciprocal manner (81). The studies reveal that S1P/S1PR1 engagement significantly promotes T-cell differentiation into Th1/Th17 while reducing into Treg phenotype.

Since the multiple regulations of S1P/S1PRs on T-cell biofunctions and responses, the blockade of S1P/S1PRs signals is regarded as a promising strategy for GVHD treatment. FTY720, derived from a metabolite of the fungus *Isaria sinclairii*, can be phosphorated by Sphk2, and the phosphorated component is defined as the functional antagonist of S1PR1 (74, 82). In the MHC-mismatched BMT mouse models, FTY720 does not impair initial donor T-cell activation and induces a rapid contraction donor T-cell pool by enhancing caspase-dependent apoptosis that ameliorated acute GVHD (56). FTY720 decreases the frequency of effector T cells by attenuating T-cell migration into the ileums and colons, not livers and lungs, and dose not trap effector T cells in secondary lymphoid organs (57). Skin is one of the major targets in both acute and chronic GVHD; sclerodermatous and skin fibrosis often occur during GVHD development after allo-HCT. Consistent with the results in acute GVHD, treatment with FTY720 early after allo-HCT restores PTEN expression and normalization of Smad3 phosphorylation and diminishes immune cell infiltration into skin, improving sclerodermatous during chronic GVHD (58). Another study also supports these findings, demonstrating that FTY720 treatment impairs CD4^+^ T cells differentiation into Th1, Th2, and Th17 and further ameliorates skin fibrosis (59). Moreover, FTY720 treatment increases IL-10 production in a subset of B cells *via* S1PR1 (57) and decreases splenic dendritic cells (CD11c^+^) by 50%, contributing to chronic GVHD control (58).

Additionally, the administration of FTY720 inhibits leukemia growth independent of S1P/S1PR signals. After CD98 internalization, FTY720 can induce rapid phosphatidylserine externalization and death of human acute myeloid leukemia (AML) cells *via* noncanonical cell death signaling (83). In chronic myelogenous leukemia (CML), protein phosphatase 2a (PP2a) activator FTY720 has been shown to process anti-leukemia activity, where synergistically genetic inhibition of BID and BIM could reverse the apoptosis induced by FTY720 (84). Moreover, FTY720 promoted toxic effects in different B-cell malignancies and B-cell from chronic lymphocytic leukemia (CLL) patients through activating PP2a and dephosphorylating of ERK1/2 (85).

Treatment involving a different S1PR1 selective agonist, CYM-5442, does not affect the proliferation and survival of donor immune cells in an MHC mismatched BMT murine model (60). However, it downregulates CCL2 and CCL7 expression in endothelial cells, reducing the migration of monocytes/macrophages into GVHD target organs and GVHD development (60). In the rat small bowel transplantation model, administration of the S1P receptor agonist W-061 significantly prevents GVHD pathogenicity in recipients by promoting donor T cells to home into the secondary lymphoid organs rather than the target organs and allografts (61).

Although FTY720 has been regarded as a promising agent for GVHD treatment, there are concerns that must be addressed. 1) In murine allo-HCT models, FTY720 treatment improves GVHD through reducing migration and inflammation and inducing apoptosis of donor T cells. FTY720 is a high-affinity agonist for all known S1PRs expect for S1PR2 while with the highest affinity for S1PR1 (82, 86). Therefore, the mice with unique S1PR conditional knockout on T cells or specific inhibitors of each S1PR should be employed to evaluate the role of individual receptor among the S1PRs in GVHD development. 2) Since a broad impact of FTY720 in S1PR signaling may cause unknown side effects, its specificity and safety need to be further determined in clinic. 3) When used at a GVHD-inhibitory dose, FTY720 does not prevent marrow engraftment and antitumor effect against T lymphoma (EL-4 cell line) in a haploidentical BMT mouse model (62), but impairs GVL effects against myeloid leukemia (C1489 cell line) in a MHC-mismatched BMT murine model (57). Thus, the GVL activity can be limited or potentially eliminated by FTY720 administration depending on tumor type post transplantation in MHC-mismatched or haploidentical mouse models (41). In human study, multiple sclerosis (MS) patients treated with FTY720 exhibit a defect in CD8^+^ T cells and subsequent anti-viral immunity, which is reflected by less IFN-γ, granzyme B production, and infiltration (87). The result would predict a negative impact of FTY720 on the GVL activity following allo-HCT in humans, although further investigation is required for validation. 4). FTY720 treatment in canine leukocyte antigen-nonidentical unrelated models does not abrogate GVHD or significantly increase survival (63). Thus, clinical translation of FTY720 after allo-HCT requires further delineation.

Furthermore, a recent study revealed that HDL infusions can significantly ameliorate the severity of GVHD through worsening immune cell infiltration and consequently attenuating both systemic and local inflammation. The authors speculated that the improved GVHD by HDL infusions may relate to S1P activity (88). Moreover, ApoM-bound S1P and HDL-bound S1P are crucial for maintaining the barrier function of epithelial cells and limiting epithelial inflammation by delivering S1P to S1PR1 (89–91). Taken together, we interpret that the S1P gradients play a dominant role in regulating both innate and adaptive immunity, which may impact the development of GVHD after allo-HCT.

### Intracellular S1P

Intracellular S1P, which can be generated by Sphk1 or Sphk2, plays a dominant role in T-cell regulation. In mouse CD8^+^ T cells, intracellular S1P produced by Sphk1 interacts with PPAR-γ to regulate lipolysis. The lack of Sphk1 can maintain central memory phenotype and higher mitochondrial respiration of CD8^+^ T cells while decreasing Treg differentiation. Pharmacological inhibition of Sphk1 using PF543 has been previously shown to promote antitumor immunity of CD8^+^ T cells (92). Transient expression of Sphk2 in T cell hybridoma augments IL-12-induced STAT4-mediated transcriptional activation (93). Mouse *Sphk2^-/-^* CD4^+^ T cells exhibit a hyperactivated phenotype with significantly enhanced proliferation and cytokine secretion in response to IL-2 and reduced sensitivity to Treg-mediated suppression *in vitro* (94). In the murine collagen-induced arthritis model, *Sphk2* knockdown increases Th1 type cytokine production and inflammation (95). Following lymphocytic choriomeningitis virus (LCMV) infection, *Sphk2^-/-^* CD4^+^ T cells displays increased activity and proliferation and promotes LCMV-specific CD8^+^ T cell responses (96). These studies demonstrate that deficiency for Sphks and intracellular S1P can induce the hyperactivated phenotypes in both CD4^+^ and CD8^+^ T cells, which may promote the occurrence and development of GVHD following allo-HCT.

In considering these comprehensive reports, we interpret that the S1P gradients in circulation would be more crucial in regulating GVHD development than the intracellular S1P under allo-HCT, the strategies specifically targeting secreted or intracellular S1P should be further determined. FTY720 was identified as a promising agent for GVHD control, even though it compromises GVL response that limits its clinical utility (**Table 1**). Therefore, a better understanding of the S1P/S1PR signaling pathway and developing specific inhibitors would provide the rationale and means to target the S1P/S1PR-signaling pathway for the benefit of patients with hematological malignancies upon allo-HCT.

There were few reports on the regulation of sphingolipid and its metabolites in the opportunistic infection post allo-HCT. Previous research showed that FTY720 treatment can prevent the homing of effector T cells to the lesions in peripheral organs while not reducing the effective priming of T- and B-cell responses in a lymphocytic choriomeningitis virus (LCMV) infected murine model (97). Therefore, we interpret that the blockade of S1P signal would not decrease the anti-infection immunity after allo-HCT. However, the impact of sphingolipid metabolism on anti-infection response after allo-HCT remains unclear. Additionally, we speculate that the administration of FTY720 other pharmacological modulators of sphingolipid metabolism may help diminishing cytokine storm and subsequently ameliorate the target organ damages caused by the breakthrough infection after allo-HCT.

## Concluding remarks

Administration of high-dose systemic steroids is still the primary treatment against GVHD, whereas steroid-refractory (SR) GVHD causes severe organ injury and significant non-relapse mortality in recipients after allo-HCT (98). A recent phase III clinical trial indicated that ruxolitinib treatment, a selective Janus kinase (JAK1 and JAK2) inhibitor, significantly improved glucocorticoid refractory acute GVHD (99). Thus, Ruxolitinib has been approved by the FDA and the European Medicines Agency for acute GvHD. Opportunistic infections and primary tumor relapse also limit wider use of HCT in the clinic. It is possible that pharmacological inhibition of sphingolipid metabolism could negatively impact immune response against infection in the patients after allo-HCT. Sphingolipid metabolism and metabolites are significantly associated with T-cell survival and function, including migratory response, differentiation and homeostasis in concert with tolerance to allografts. This review summarized published findings and discussed the regulation of sphingolipid metabolism and metabolites with respect to T-cell response. We also presented therapeutic strategies that target sphingolipid metabolism and help reduce GVHD while preserving GVL activity. Further investigation regarding how sphingolipids impact T-cell alloreactivity and anti-leukemia immunity and development of specific pharmacological inhibitors are highly warranted avenues of future work in the field as these would improve clinical immunotherapy and eventually benefit patients with hematologic malignancies.

## Author contributions

LT drafted the manuscript and LT, X-ZY, BC and BO revised the manuscript. All authors contributed to the article and approved the submitted version.

## Funding

This work was supported in part by research funding from the National Institutes of Health including R01 HL140953, R01 CA258440 and R21 CA263140 (X-ZY), and R01 CA214461, R01 DE016572, and P01 CA203628 (BO).

## Acknowledgments

We thank the current and former members in Yu Lab who contribute to the related work. We are also grateful for Dr. Roger Johnson who provided critical comments for the manuscript.

## Conflict of interest

The authors declare that the research was conducted in the absence of any commercial or financial relationships that could be construed as a potential conflict of interest.

## Publisher’s note

All claims expressed in this article are solely those of the authors and do not necessarily represent those of their affiliated organizations, or those of the publisher, the editors and the reviewers. Any product that may be evaluated in this article, or claim that may be made by its manufacturer, is not guaranteed or endorsed by the publisher.
